# Microstructure and Optoelectronic Properties of WZO/Al/Cu/Al/WZO Multilayer Films

**DOI:** 10.3390/nano15221711

**Published:** 2025-11-12

**Authors:** Haijuan Mei, Liying Liu, Qingfeng Zhu, Huojuan Ye, Zhenting Zhao, Qiuguo Li, Jicheng Ding, Yi Yu, Libin Gan, Yuhang Li, Jie Liu, Weiping Gong

**Affiliations:** 1 Guangdong Provincial Key Laboratory of Electronic Functional Materials and Devices, Huizhou University, Huizhou 516007, China; haijuanmei@hzu.edu.cn (H.M.); clara654@163.com (L.L.); 13823945708@163.com (H.Y.); zhzhting@hzu.edu.cn (Z.Z.); liqiuguo10@163.com (Q.L.); xqf20081213@126.com (Y.Y.); glb945896652@163.com (L.G.); liyuhang02171030@163.com (Y.L.); 2 School of Materials Science and Engineering, Anhui University of Technology, Maanshan 243002, China; jcdingxinyang@126.com; 3 School of Intelligent Manufacturing, Guangzhou Polytechnic University, Guangzhou 511483, China

**Keywords:** WZO/Al/Cu/Al/WZO, Cu layer thickness, microstructure, optoelectronic properties

## Abstract

By adjusting the Cu layer thickness, this study systematically investigated the evolution of the microstructure and optoelectronic properties of WZO/Al/Cu/Al/WZO multilayer films. The results indicated that all the films exhibited a ZnO phase with hexagonal wurtzite structure and a Cu phase with face-centered cubic structure, showing preferred orientations along the (002) and (111) planes, respectively. As the Cu layer thickness increased from 5 nm to 13 nm, its crystallinity was substantially improved, with the grain size gradually increasing from 4.7 nm to 12.4 nm. In contrast, the crystalline quality of ZnO first improved and then deteriorated, reaching an optimum at a Cu layer thickness of 7 nm. With increasing the Cu layer thickness, the visible light absorption loss was enhanced and then resulted in a gradual decrease in transmittance from 79.2% to 68.0%. Benefiting from the significant improvement in the crystallinity and continuity of the Cu layer, the resistivity sharply decreased from 1.7 × 10^−3^ Ω·cm to 7.1 × 10^−5^ Ω·cm and tended to saturate when the thickness exceeded 9 nm. As the Cu thickness increased to 11 nm, the figure of merit (*F_OM_*) reached a maximum value of 4.4 × 10^−3^ Ω^−1^, demonstrating the optimal optoelectronic performance.

## 1. Introduction

Owing to their excellent combination of high visible light transmittance and high electrical conductivity, transparent conductive oxides (TCO) have become an integral component of modern optoelectronic devices, showing broad application prospects in fields such as solar cells, flat-panel displays, and organic light-emitting diodes [1,2,3,4]. Due to its excellent optoelectronic properties, tin-doped indium oxide (ITO) has long held a dominant market position and has become the most commercially mature TCO film [5,6,7]. However, the broader application of ITO film is currently confronted with two significant challenges. Firstly, its core constituent, indium, is a rare and expensive metal, resulting in high raw material costs. Secondly, the inherent brittleness of ITO film leads to poor adhesion on flexible substrate and weak resistance to bending. This fundamental defect severely restricts their potential for application in emerging fields such as flexible electronics and wearable devices.

As a typical n-type wide-bandgap semiconductor, ZnO is regarded as one of the most promising candidates to replace ITO, owing to its abundant raw material reserves, low cost, environmental non-toxicity, and excellent flexibility. However, the pure ZnO film exhibits poor electrical conductivity due to its low carrier concentration, which limits its direct application. To overcome this limitation, element doping has become a core strategy for enhancing optoelectronic properties, such as Al-doped ZnO (AZO) [8], Ga-doped ZnO (GZO) [9], F-doped ZnO (FZO) [10], and W-doped ZnO (WZO) [11]. Among the various doping elements, W, as a transition metal with a high valence state, exhibits unique doping advantages. When the W^6+^ replaced the Zn^2+^ with similar ionic radius, it not only significantly increased the carrier concentration by providing more free electrons but also avoided inducing significant lattice distortion [12,13,14,15]. However, compared with the commercial ITO film, the resistivity of WZO film is still high, which limits its further application in high-performance optoelectronic devices.

To further enhance the comprehensive optoelectronic performance of TCO films, a TCO/metal/TCO (TMT) multilayer structure has been proposed. This structure embeds an ultra-thin metal layer between two TCO dielectric layers, utilizing the high electrical conductivity of the metal layer to significantly reduce the sheet resistance of the film. Concurrently, the TCO layers on both sides effectively suppress the reflection of the metal layer through optical interference effects, achieving high transmittance in the visible light and thereby synergistically optimizing the optoelectronic performance of the films [16,17,18]. However, Ag or Cu atoms are prone to oxidation and diffusion at high temperatures, leading to a sharp deterioration in the optoelectronic performance of the TMT multilayer films. To improve the high-temperature stability of the films, ultra-thin layers such as Al, Ti, or Ni are typically introduced to construct four- or five-layer structures, such as AZO/Ni/Ag/AZO [19], AZO/Ti/Ag/AZO [20], AZO/Ti/Cu/AZO [21,22], and AZO/Al/Ag/Al/AZO [23]. This interfacial layer can be used as a physical barrier layer and diffusion barrier, while improving the interlayer adhesion, thus significantly enhancing the thermal and temporal stability of the films. In the multilayer structure, the selection and thickness control of the metal layer are crucial for determining its final performance. Copper, owing to its low cost and high electrical conductivity, has become an attractive material for the metal layer. However, the thickness of the Cu layer plays a decisive role in the performance. An overly thin Cu layer exhibited poor crystallinity, making it difficult to form a continuous and uniform conductive network, and then resulted in a higher sheet resistance. Conversely, an overly thick Cu layer caused a sharp decline in the visible light transmittance of the films due to strong intrinsic absorption and light reflection [24,25,26]. Thus, precisely optimizing the thickness of Cu layer to find the optimal balance between electrical conductivity and optical transmittance is key to preparing high-performance TMT films.

Although the influence of Cu layer thickness on the optoelectronic properties of TMT multilayer films has been extensively studied, its specific functional mechanism within the five-layer WZO/Al/Cu/Al/WZO structure remains to be further elucidated. In this study, the WZO/Al/Cu/Al/WZO films were designed and fabricated, the influence of Cu layer thickness on the microstructure and optoelectronic properties of the films was systematically investigated.

## 2. Experimental Details

### 2.1. Coating Deposition

**Figure 1:**
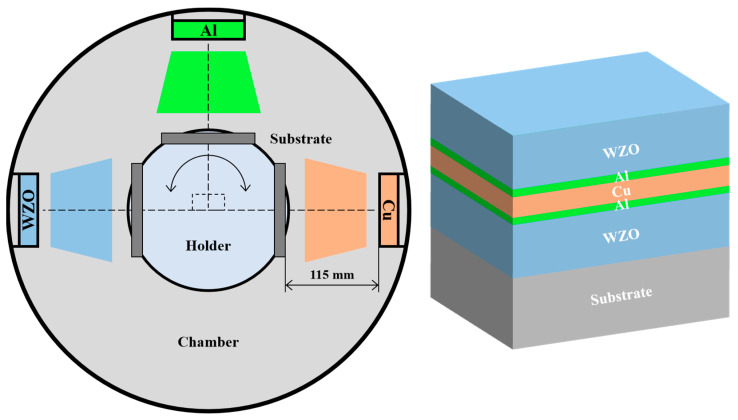
Schematic diagram of the sputtering deposition system and multilayer structure.

WZO/Al/Cu/Al/WZO multilayer films were deposited on soda-lime glass substrates (20 × 20 × 0.4 mm^3^) by DC and RF magnetron sputtering using the ceramic WZO target (99.99% purity, ZnO:W = 98:2 wt%, Ø76.2 × 4 mm^2^), metal Al and Cu targets (99.99% purity, Ø76.2 × 4 mm^2^). Figure 1 presents the schematic diagram of the alternating deposition system employed. A rotational substrate holder with a rotation speed of 5 rpm was placed in the chamber, the target-substrate distance was set at 115 mm. All the substrates were ultrasonically cleaned in acetone and alcohol for 20 min, respectively, and then placed on a rotating substrate holder subsequent to desiccation. Prior to deposition, the vacuum chamber was pumped to 8.0 × 10^−4^ Pa, and heated to 200 °C. To eliminate surface contaminants, all the substrates were bombarded by Ar^+^ ions at a high bias voltage of 800 V for 15 min. The deposition process was initiated by introducing high-purity argon (Ar) gas into the chamber, establishing a working pressure of 0.5 Pa. Both the bottom and top WZO layers were fabricated via RF magnetron sputtering with a ceramic WZO target. The sputtering was conducted for 12 min at a target power of 70 W. Subsequently, a thin Al layer was deposited immediately before and after the Cu layer using RF magnetron sputtering. This sputtering was performed at a target power of 300 W for a duration of 5 s. Then the Cu interlayer was deposited by DC magnetron sputtering at a target power of 30 W. To investigate the effect of Cu thickness, the deposition time was varied from 33 to 85 s, yielding Cu layer thicknesses ranging from 5 to 13 nm. A complete list of the deposition parameters is provided in Table 1.

### 2.2. Coating Characterization

The cross-section morphologies and the thicknesses of the films were examined using a scanning electron microscope (SEM, Tescan Vega 3 Xmu, Brno, Czech Republic). The surface roughness was examined over a scan area of 8 × 8 μm^2^ by using an atomic force microscope (AFM, MFP-3D, Oxford, MS, USA) in contact mode. The phase structure was analyzed by X-ray diffraction (XRD, Bruker D8 advance, Karlsruhe, Germany) equipped with a Cu *K_α_* radiation source. The residual stress, lattice parameter, and grain size were calculated from XRD data based on the biaxial strain model [27], Bragg’s law [24], and Scherrer equation [28], respectively.

(1) $$\sigma \;=-233\times \frac{c-c_{0}}{c_{0}}$$

(2) $$c\;=\;\frac{\lambda \;}{\sin\theta \;}$$

(3) $$D\;=\;\frac{0.9\lambda \;}{\beta \cos\theta \;}$$

where λ, β, and *c*_0_ = 5.2066 Å refer to the wavelength of Cu *K_α_* radiation, the full width at half maximum, and the unstrained lattice constant of ZnO along the c-axis, respectively. The microstructure was further characterized by transmission electron microscopy (TEM, Talos F200X, Thermo, Waltham, MA, USA). The transmittance was measured over the wavelength range of 300 to 1000 nm using by a visible spectrophotometer (723PCSR, Ruifeng, Guangzhou, China). Based on the van der pauw principle, electrical resistivity, carrier concentration, and mobility were determined using a Hall effect measurement system (CH-100, Cuihai, Beijing, China).

## 3. Results

### 3.1. Microstructure

**Figure 2:**
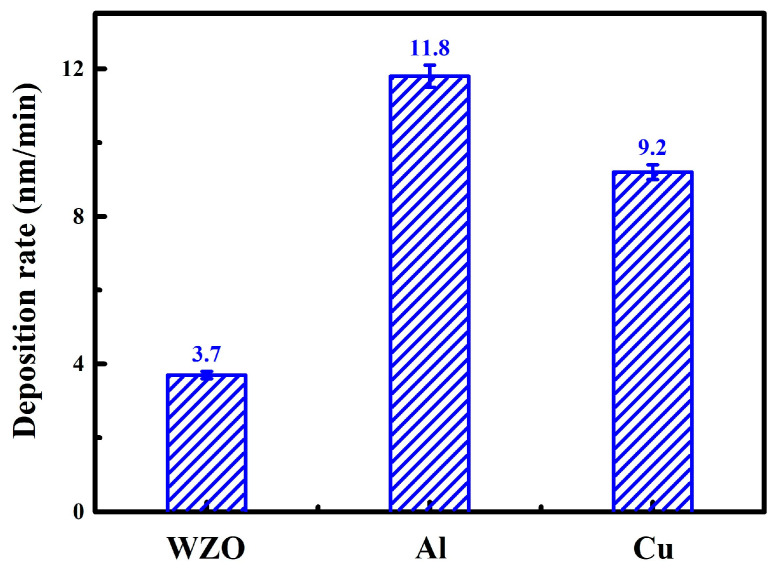
Deposition rate of the monolayer films.

Figure 2 presents a comparison of the deposition rates for the WZO, Al, and Cu single-layer films under specific sputtering process parameters. When the target powers were set at 70 W, 300 W, and 30 W, respectively, the deposition rates of the three films were 3.7 nm/min, 11.8 nm/min, and 9.2 nm/min, exhibiting significant differences. This phenomenon revealed the complex synergistic regulation mechanism among the sputter yield of the target material, its intrinsic physical properties, and the sputtering process parameters. Compared to the Al and Cu metallic films, the WZO film exhibited the lowest deposition rate, which was primarily attributed to its inherent properties as a composite oxide ceramic material. Firstly, as a composite oxide, WZO possessed a low sputter yield and high atomic binding energy, which increased the energy threshold required for the target atoms to be effectively sputtered and ejected from the surface. Secondly, being a ceramic target, WZO had poor electrical conductivity. During the sputtering process, this easily led to charge accumulation on the target surface, causing unstable plasma discharge and reducing the energy coupling efficiency, which further limited the deposition rate. In contrast, although the Al metallic film did not have the highest sputter yield, it achieved a high deposition rate under the drive of a high power of 300 W, which provided high incident ion energy and flux. Meanwhile, due to its dual advantages of high sputter yield and excellent electrical conductivity, the Cu metallic film still achieved efficient deposition even at a low target power of 30 W.

Based on the optimization of deposition process parameters and the precise control of deposition time, the thickness of the multilayer films could be precisely controlled. As shown in Table 2, the thicknesses of the WZO and Al layers were fixed at 44 nm and 1 nm, respectively, the total thickness of the films correspondingly increased from 95 nm to 103 nm as the Cu layer thickness was gradually increased from 5 nm to 13 nm. In this structure, WZO and Cu were used as high-refractive-index dielectric layers and highly reflection metallic layer, respectively. While Al was used as interface layer to effectively adjust the transmittance of the multilayer film. Based on prior optimization experiments and literature [20], the multilayer film exhibited optimal optoelectronic properties when the Al layer thickness was 1 nm. In addition, according to the principle of destructive interference, the WZO layer thicknesses were optimized so that when the total thickness of the films approached 100 nm, the light beams reflected from the different interfaces satisfied the conditions for destructive interference, thus minimizing the reflectance and maximizing the transmittance of visible light.

**Figure 3:**
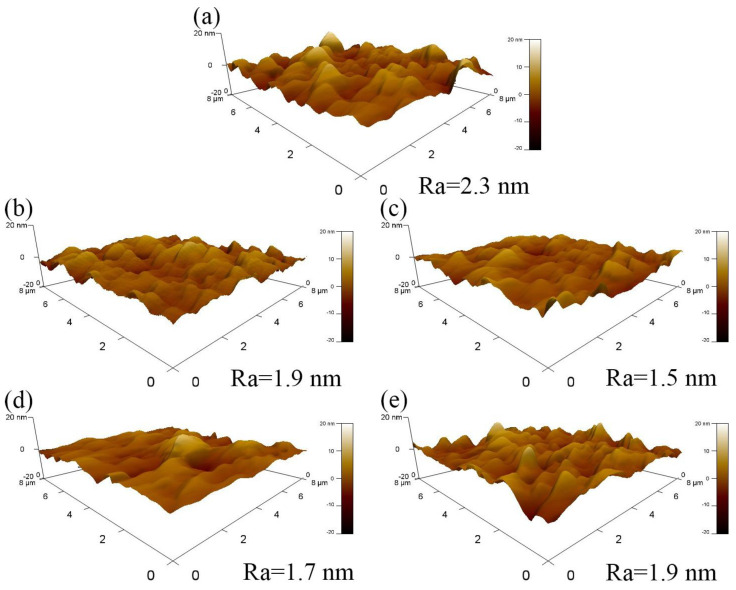
AFM images of WZO/Al/Cu/Al/WZO films at various Cu thicknesses: (**a**) 5 nm, (**b**) 7 nm, (**c**) 9 nm, (**d**) 11 nm, (**e**) 13 nm.

Figure 3 presents the AFM images of the WZO/Al/Cu/Al/WZO multilayer films. As the Cu layer thickness increased, the surface roughness of the films first decreased and then increased. When the Cu layer thickness increased from 5 nm to 9 nm, the surface roughness gradually decreased from 2.3 nm to 1.5 nm. As the Cu layer thickness further increased, the surface roughness then gradually rebounded to 1.9 nm. This phenomenon was closely related to the crystallization behavior and microstructural evolution of the Cu layer. In the initial stage, when the Cu layer was thin, its growth followed an island growth mode, where incomplete nucleation led to the formation of discrete island-like structures, which resulted in a relatively rough surface [29]. When the Cu layer thickness increased, its crystallinity improved, and it gradually transitioned from discrete islands to a continuous and dense film, thereby reducing the surface roughness [24]. However, when the Cu layer thickness exceeded a certain critical value, the excessive growth and coarsening effect of the grains began to dominate, and the formation of coarse grains introduced new surface undulations, leading to the increase in surface roughness [30].

**Figure 4:**
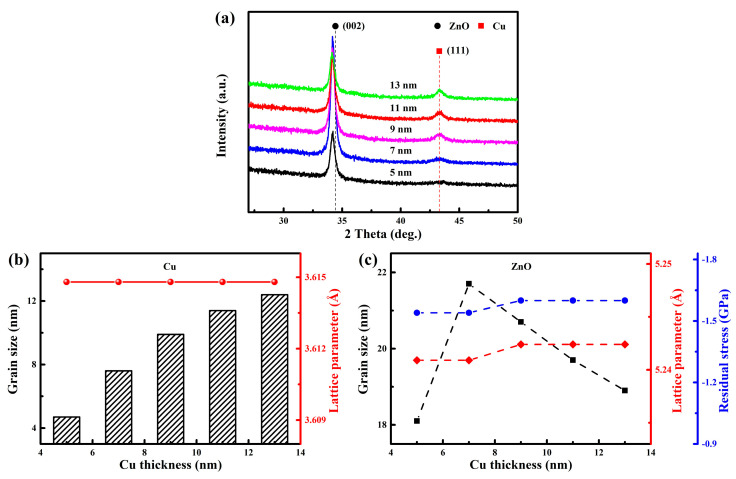
(**a**) XRD pattern, (**b**) grain size and lattice parameter of the Cu films, (**c**) grain size, lattice parameter, and residual stress of the WZO films.

Figure 4 presents the XRD patterns, grain sizes, lattice constants, and residual stress of the WZO/Al/Cu/Al/WZO multilayer films. As shown in Figure 4a, a diffraction peak from the (002) plane of the ZnO phase was detected at approximately 34.2°, indicating that the WZO film showed a hexagonal wurtzite structure with a preferential orientation along the c-axis. Compared to the standard diffraction peak of ZnO (JCPDS 36-1451), the (002) diffraction peak was shifted to a lower angle, which was primarily attributed to the existence of residual stress within the film [11]. In addition, a weak diffraction peak was observed at around 43.3°, corresponding to the (111) crystal plane of the Cu phase with a face-centered-cubic structure and a lattice parameter of 3.615 Å. With increasing the Cu layer thickness, the intensity of the (111) diffraction peak gradually enhanced, indicating that the crystalline quality of the Cu film was gradually improved. As shown in Figure 4b, as the Cu layer thickness increased from 5 nm to 13 nm, the grain size significantly increased from 4.7 nm to 12.4 nm. From the perspective of thin-film growth kinetics, this phenomenon could be attributed to the evolution of the growth mode. The increase in thickness prompted the film to transition from an initial island growth mode to a more continuous columnar crystal structure. During this transition, the surface mobility of the deposited atoms was enhanced, which facilitated the formation of larger grains with lower defect densities. This microstructural evolution was macroscopically manifested as an enhancement in the diffraction peak intensity and an increase in grain size, which finally led to a significant improvement in the crystalline quality of the Cu film [31].

As Cu layer thickness increased, the ZnO (002) diffraction peak shifted to a lower angle by approximately 0.01°. According to Bragg’s law, this low-angle shift directly corresponded to an increase in the interplanar spacing, which indicated an expansion of the c-axis lattice constant. As shown in Figure 4c, both the residual stress and lattice constant of the WZO film showed a slight upward trend with increasing the Cu layer thickness. This demonstrated that the Cu layer served not only as a conductor, but also as a stress-regulating and structural-modifying layer, altering the lattice constant of ZnO through mechanisms such as interfacial strain and stress relaxation. Furthermore, as the Cu layer thickness increased, the ZnO grain size exhibited a trend of first increasing and then decreasing. When the Cu layer thickness increased from 5 nm to 7 nm, the intensity of the (002) diffraction peak increased sharply, and the ZnO grain size increased from 18.1 nm to 21.7 nm. This indicated a substantial enhancement in the crystallinity of the WZO film, which can be related to the evolution of the growth mode of Cu layer. At thinner thicknesses, the Cu layer tended to follow an island growth mode, forming discrete island-like structures. This discontinuous island-like structure has high surface energy and large defect density, could not provide a smooth, low-defect nucleation substrate for the growth of the top WZO film, thereby severely restricting the nucleation and growth of ZnO grains, resulting in poor crystalline quality. When the Cu layer thickness was increased to 7 nm, its own continuity improved and its crystallinity was significantly enhanced, and the growth mode gradually transformed from discrete island-like structure to a continuous growth mode. This high-quality and continuous Cu layer provided a more ideal template for the growth of the top WZO layer, effectively reduced the interfacial energy, and promoted the preferential orientation growth of ZnO grains, which in turn significantly enhanced its overall crystallinity [31]. However, as the Cu layer thickness further increased, the intensity of the (002) diffraction peak began to decrease, and the ZnO grain size gradually decreased to 18.9 nm. This indicated that the deterioration of crystallization quality of the WZO film, which could be attributed to the accumulated lattice defects and the inherent internal stress within the Cu layer. These defects and stresses disrupted the smoothness and periodicity of the Cu layer surface, and then exacerbated the lattice mismatch and interfacial stress between the Cu and top WZO layers, thus inhibiting the further growth of ZnO grains, and finally leading to a decline in crystallinity [30]. Similar phenomenon was also reported in AZO/Cu/AZO multilayer films [24,32], the ZnO grain size reached the maximum value and exhibited the highest crystalline quality when the Cu layer thickness was increased to 7 nm.

**Figure 5:**
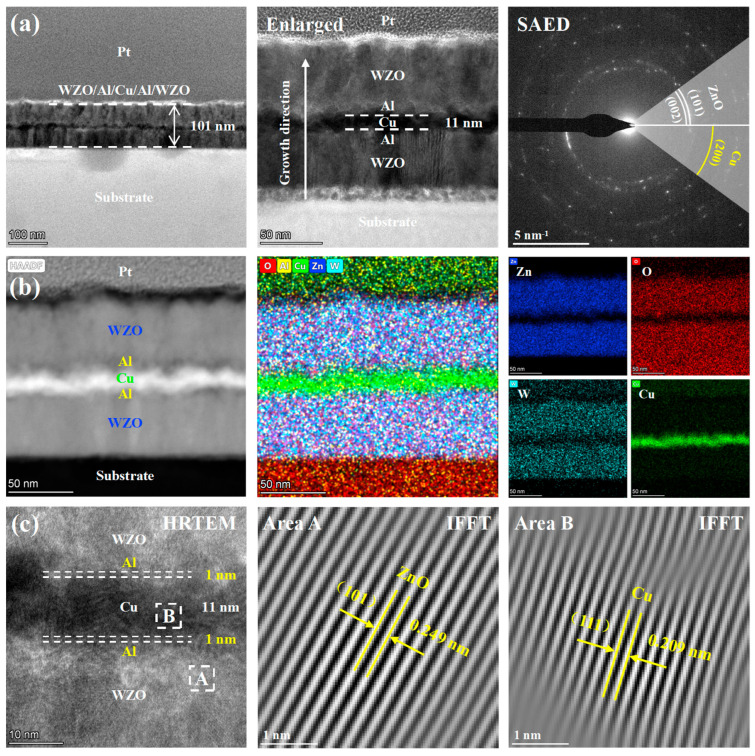
TEM images of the WZO/Al/Cu (11 nm)/Al/WZO film: (**a**) Bright-field images and SAED pattern, (**b**) HAADF image and STEM mapping, (**c**) HRTEM image and IFFT pattern.

The microstructure and nano-multilayer structure of the WZO/Al/Cu (11 nm)/Al/WZO film were systematically analyzed by TEM. As shown in the cross-sectional bright-field image in Figure 5a, the total thickness of the film was approximately 101 nm, and its interior exhibited a periodic multilayer structure that was in high agreement with the design. The contrast difference in the image primarily originated from the different atomic numbers of the elements in each layer. The Cu layer, with a higher atomic number, appeared as a relatively dark area due to its stronger electron scattering capability, and its thickness was precisely controlled at 11 nm, consistent with the designed value. The SAED analysis further indicated that the film exhibited a polycrystalline structure. The discontinuous diffraction rings in diffraction pattern were referred to the hexagonal wurtzite ZnO phase and the face-centered-cubic Cu phase, respectively, which was consistent with the XRD results. However, owing to the thin thickness of the Al layer (1 nm), its diffraction signal was not detected in the SAED pattern, suggesting that the Al layer might exist in an amorphous form or as nanocrystals in the interfacial regions. As shown in Figure 5b, the HAADF and STEM mapping images clearly demonstrated the multilayer structure and the interfaces between layers. The elemental distribution within each functional layer was uniform, the interfaces were sharp, and no significant elemental interdiffusion was observed, indicating that a highly controlled, layer -by-layer growth was achieved. However, the STEM mapping for Al layer was compromised by the extremely thin nature of the thickness. In Figure 5c, the HRTEM image, combined with the IFFT (Inverse Fast Fourier Transform) pattern, clearly identified the (101) plane of ZnO and the (111) plane of Cu phase. The interplanar spacings were measured to be 0.249 nm and 0.209 nm, respectively, which were in high agreement with the theoretical values of the standard interplanar spacings of ZnO and Cu phases.

### 3.2. Optoelectronic Properties

**Figure 6:**
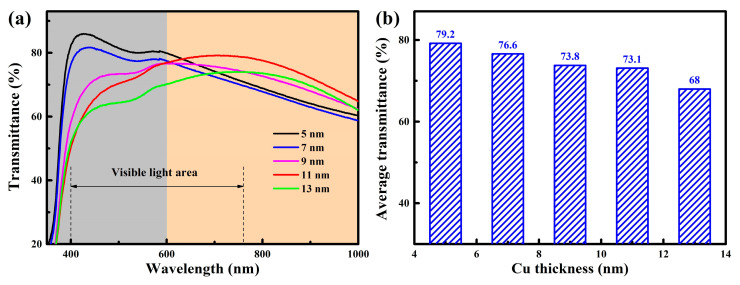
(**a**) Transmittance spectra, (**b**) average transmittance of the WZO/Al/Cu/Al/WZO films.

Figure 6 shows the transmittance spectra and average transmittance of the films. As shown in Figure 6a, in the short-wavelength spectral region (350–600 nm), the average transmittance of the films gradually decreased as the Cu layer thickness increased. This was mainly attributed to the enhanced absorption and reflection effects caused by the increasing thickness of the Cu layer, which suppressed light transmission. However, in the long-wavelength spectral region (600–1000 nm), the average transmittance exhibited a non-monotonic trend, first decreasing, then increasing, and subsequently decreasing again, reaching its maximum at a Cu layer thickness of 11 nm. A similar phenomenon was also reported in the AZO/Cu/AZO multilayer films [31]. When the Cu layer reached a certain critical thickness, its optical behavior approached that of a mirror, while the multilayer structure effectively suppressed the reflection of Cu layer through destructive interference, thereby enhancing the overall transmittance of the films [24]. Furthermore, with the increase in Cu layer thickness, the continuity and crystallinity of Cu film were significantly improved, the grain size gradually increased, and then the grain boundary density decreased, which reduced the grain boundary scattering and further improved the transmittance [33]. In Figure 6b, within the visible spectral region (400–760 nm), the average transmittance of the films exhibited a progressively decreasing trend. When the Cu layer thickness increased from 5 nm to 13 nm, the average transmittance of the films gradually decreased from 79.2% to 68.0%. Although the increase in Cu grain size helped to reduce the grain boundary scattering, the intrinsic absorption effect of the Cu layer was dominant in the visible region. As the Cu layer thickness increased, its absorption loss as a metal layer to visible light was significantly enhanced, which eventually led to the decrease of overall transmittance [24].

**Figure 7:**
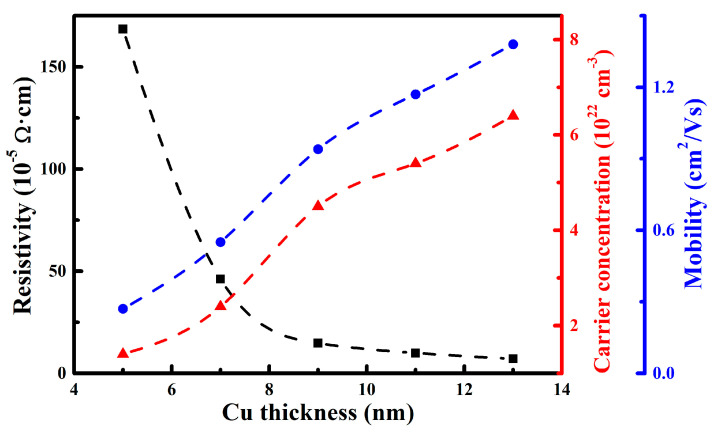
Resistivity, carrier concentration, and mobility of the WZO/Al/Cu/Al/WZO films.

Figure 7 displays the resistivity, carrier concentration, and Hall mobility of the films. With the increase in Cu layer thickness, the carrier concentration and Hall mobility of the films gradually increased from 1.4 × 10^22^ cm^−3^ and 0.3 cm^2^/V·s to 6.4 × 10^22^ cm^−3^ and 1.4 cm^2^/V·s, respectively, and then the resistivity exhibited a decreasing trend. Specifically, as the Cu layer thickness increased from 5 nm to 9 nm, the resistivity sharply decreased by one order of magnitude, from 1.7 × 10^−3^ Ω·cm to 1.5 × 10^−4^ Ω·cm. This phenomenon was attributed to the significant improvement in the crystallinity and continuity of the Cu layer, which was characterized by a microstructural evolution from discrete islands to a continuous and dense film [34]. In the initial stage, the thin Cu film followed an island growth mode, forming some discontinuous and isolated crystalline islands. As the main electron scattering centers, these islands seriously hindered the effective transport of carriers, resulting in low mobility. When the Cu layer thickness increased to 9 nm, the island-like structures began to coalesce, forming continuous conductive pathways, and the crystalline quality was also significantly improved. This structural transition directly led to a sharp increase in both carrier concentration and Hall mobility, thereby significantly reducing the resistivity of the films. With the further increase in Cu layer thickness, the decreasing trend of the resistivity gradually slowed, indicating that the electrical conductivity was approaching saturation. Based on above XRD analysis results, the continuity and crystallinity of the Cu film were well-developed when the Cu layer thickness exceeded 9 nm. As the core conductive layer of the multilayer film, a further increase in the Cu layer thickness primarily promoted grain growth. However, once the grain size reached a certain limit, its growth rate decelerated. Consequently, the potential for further improvement in carrier mobility, which was dominated by grain boundary scattering, became smaller. This led to a corresponding slowdown in the growth rates of carrier concentration and Hall mobility, ultimately leading to the gradual stabilization of the overall conductivity enhancement. When the Cu layer thickness increased to 13 nm, the WZO/Al/Cu/Al/WZO multilayer film achieved the lowest resistivity of 7.1 × 10^−5^ Ω·cm. This value was significantly lower than the resistivity of 2.1 × 10^−4^ Ω·cm reported for the AZO/Cu (13 nm)/AZO film [24]. This superior performance was primarily attributed to the higher crystallinity of the Cu layer prepared in this study, which effectively reduced the scattering of electrons by grain boundaries. In addition, the measured Hall coefficient was negative, implying that the carrier type was electron, namely N-type semiconductor.

**Figure 8:**
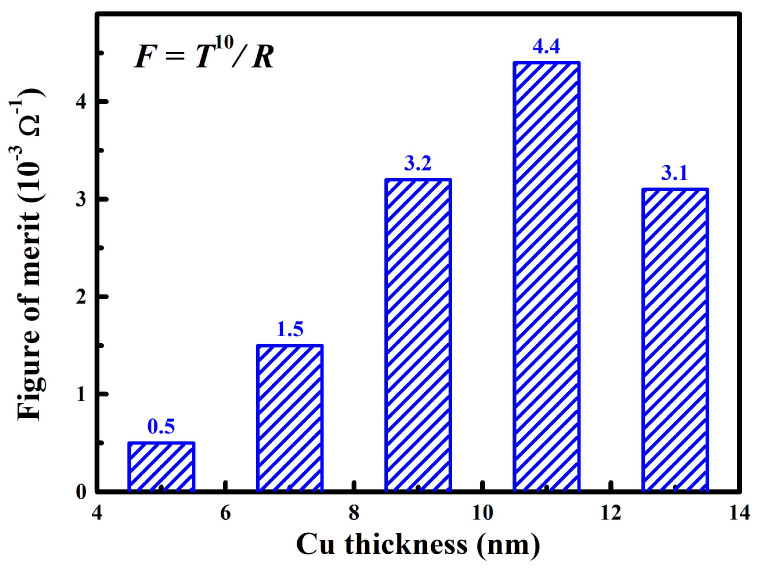
Figure of merit of the WZO/Al/Cu/Al/WZO films at various Cu thicknesses.

The figure of merit (*F_OM_*) is a critical parameter for the comprehensive evaluation of the optoelectronic performance of transparent conductive films, as its physical essence lies in quantifying the trade-off between optical transmittance and electrical conductivity [35], which can be defined as follows:

(4) $$F_{OM}\;=\frac{T}{R}$$

where *T* and *R* referred to the average transmittance and sheet resistance, respectively. Figure 8 clearly reveals the variation of *F_OM_* for the WZO/Al/Cu/Al/WZO films with the increase in the Cu layer thickness, which exhibited a typical non-monotonic trend of first increasing and then decreasing. This indicated the existence of an optimal Cu layer thickness for achieving the best balance of optoelectronic properties. When the Cu layer thickness increased from 5 nm to 11 nm, the *F_OM_* gradually improved from 5.0 × 10^−4^ Ω^−1^ to the maximum value of 4.4 × 10^−3^ Ω^−1^, demonstrating the best comprehensive optoelectronic performance. The significant enhancement can be related to the microstructure evolution of the Cu layer. As discussed above, with increasing thickness, the Cu layer transformed from a discontinuous island structure into a continuous film with excellent crystallinity, which greatly optimized its function as the core conductive layer. The formation of a continuous film established an efficient carrier transport network, leading to substantial increases in carrier concentration and Hall mobility, and a consequent sharp decrease in the resistivity. At this stage, the gain in electrical conductivity far exceeded the minor loss in optical transmittance, thus dominating the trend of the *F_OM_*. However, when the Cu layer thickness was further increased to 13 nm, the *F_OM_* showed a downward trend. This phenomenon indicated that although the increasing thickness continued to improve the crystallinity of Cu layer and reduce resistivity, the effects of absorption and reflection of visible light by the Cu layer had gradually become the dominant factors. Once the thickness of Cu layer exceeded a certain critical value, its photon absorption coefficient increased dramatically, causing a significant degradation in transmittance, and then led to the deterioration of the overall optoelectronic performance [31].

## 4. Conclusions

In this study, WZO/Al/Cu/Al/WZO films were successfully fabricated on glass substrates by an alternative deposition system using DC and RF magnetron sputtering. The influence of Cu layer thickness on the microstructure and optoelectronic properties of the films was systematically investigated. The main conclusions are as follows:

(1) All the films were composed of a hexagonal wurtzite ZnO phase with a strong (002) preferred orientation and a face-centered cubic Cu phase with a (111) preferred orientation. As the Cu layer thickness increased from 5 nm to 13 nm, both the intensity of the (111) diffraction peak and grain size increased significantly, leading to a marked improvement in the crystallinity. Notably, the crystalline quality of ZnO was optimized at a Cu layer thickness of 7 nm.
(2) With increasing the Cu layer thickness, its absorption loss as a metallic layer in the visible region was gradually enhanced, causing a decrease in the transmittance from 79.2% to 68.0%. Due to a significant improvement in the crystallinity and continuity of the Cu layer, the resistivity of the films decreased sharply from 1.7 × 10^−3^ Ω·cm to 7.1 × 10^−5^ Ω·cm, and the decreasing trend tends to be flat after the Cu thickness exceeded 9 nm.
(3) When the Cu layer thickness increased to 11 nm, the film achieved an optimal balance between transmittance and electrical conductivity, the *F_OM_* reached a maximum value of 4.4 × 10^−3^ Ω^−1^, exhibiting the best comprehensive optoelectronic properties.

## Figures and Tables

**Table 1:** Deposition parameters of WZO/Al/Cu/Al/WZO films.

Parameters			
Base pressure (Pa)	8.0 × 10^−4^		
Working temperature (°C)	200		
Working pressure (Pa)	0.5		
Target to substrate distance (mm)	115		
Target material	WZO	Al	Cu
Magnetron sputtering	RF	RF	DC
Target power (W)	70	300	30
Deposition time	12 min	5 s	33, 46, 59, 72, 85 s

**Table 2:** Monolayer thickness and total thickness of WZO/Al/Cu/Al/WZO films.

Monolayer Thickness (nm)					Total Thickness (nm)
WZO	Al	Cu	Al	WZO	
44	1	5	1	44	95
44	1	7	1	44	97
44	1	9	1	44	99
44	1	11	1	44	101
44	1	13	1	44	103

## Data Availability

Data are contained within the article.
